# Clinicians’ perspective on the implemented KLIK PROM portal in clinical practice

**DOI:** 10.1007/s11136-020-02522-5

**Published:** 2020-05-28

**Authors:** Lorynn Teela, Maud M. van Muilekom, Lieke H. Kooij, Anouk W. Gathier, Johannes B. van Goudoever, Martha A. Grootenhuis, Lotte Haverman, Hedy A. van Oers

**Affiliations:** 1 Psychosocial Department, Emma Children’s Hospital, Amsterdam UMC, University of Amsterdam, G8-136, Meibergdreef 9, Postbox 22660, 1100 DD Amsterdam, The Netherlands; 2 Department of Pediatrics, Emma Children’s Hospital, Amsterdam UMC, University of Amsterdam, Vrije Universiteit Amsterdam, Meibergdreef 9, Amsterdam, The Netherlands; 3 Princess Máxima Center for Pediatric Oncology, Utrecht, The Netherlands

## Abstract

**Purpose:**

Since 2011, the evidence-based KLIK Patient Reported Outcome Measure (PROM) portal has been implemented in clinical practice in > 20 Dutch hospitals. Patients and/or parents complete PROMs on Health Related Quality of Life, symptoms and psychosocial functioning before their outpatient consultation. Answers are converted into an ePROfile and discussed by clinicians during consultation to monitor well-being over time and detect problems early. This study aims to get insight into the KLIK implementation from the clinician’s perspective.

**Methods:**

As part of the KLIK implementation process, annual meetings were held with multidisciplinary teams to evaluate the use of KLIK. An online questionnaire was sent regarding (1) overall satisfaction, (2) feeling competent to discuss PROMs, (3) use of KLIK during the consultation, (4) influence of KLIK on the consultation, (5) usability of the KLIK PROM portal, (6) satisfaction with PROMs and feedback, and (7) support of the KLIK expert team. Open questions about (dis)advantages were included. Descriptive analyses were used.

**Results:**

One hundred and forty-eight clinicians (response-rate 61%) from 14 hospitals in the Netherlands participated. Results show that: (1) clinicians report an overall satisfaction of median = 69/100 (visual analogue scale), (2) 85.8% feel competent discussing the ePROfile, (3) 70.3% (almost) always discuss the ePROfile, (4) 70.3% think that KLIK improves consultation, (5) 71.6% think KLIK is easy to use, (6) 80.4% are satisfied with the feedback of the overall KLIK ePROfile, (7) 71.6% experience sufficient support of the KLIK team.

**Conclusion:**

Participating clinicians are generally satisfied with KLIK. Improvements to the KLIK PROM portal are now realized based on the mentioned disadvantages (e.g., shorten PROM completion by use of PROMIS and integrating KLIK with Electronic Health Records).

**Electronic supplementary material:**

The online version of this article (10.1007/s11136-020-02522-5) contains supplementary material, which is available to authorized users.

## Introduction

In the past decades, there has been increased attention for the use of Patient Reported Outcome Measures (PROMs) in daily clinical practice enabling patient-centered care [1]. Discussing PROMs in the consultation room empowers patients, enhances patient-clinician communication, and promotes shared decision making [2–5]. Monitoring patients by using PROMs increases awareness for patients’ concerns, facilitates recognition of physical or psychological problems, improves patient satisfaction with health care, and is associated with improved treatment outcomes, including survival [3, 4, 6–8].

**Fig. 1:**
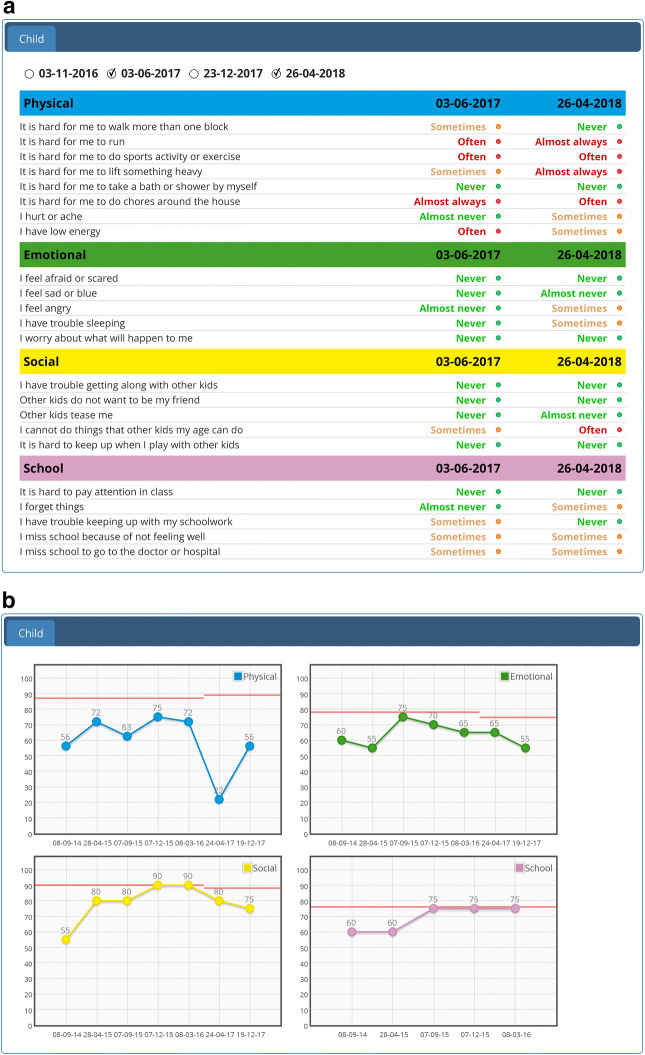
**a** KLIK ePROfile - literal feedback of the individual items on the Pediatric Quality of Life Inventory (PedsQL) **b** KLIK ePROfile - graphical feedback of the PedsQL, including norm lines

After two efficacy studies [9, 10], the KLIK PROM portal (www.hetklikt.nu) is being implemented in daily clinical practice since 2011. These studies showed that the feedback and discussion of PROMs in the consultation room resulted in more attention for, and improved identification of, psychosocial and emotional problems and increased satisfaction of pediatricians with the provided care [9, 10]. Within the KLIK PROM portal, pediatric patients (≥ 8 years) and/or their parents and adult patients are asked to complete PROMs regarding Health Related Quality of Life (HRQOL), symptoms and/or psychosocial functioning online at home prior to the outpatient consultation. The answers are converted into an electronic PROfile (KLIK ePROfile, Fig. 1) that contains a broad range of feedback options tailored to each specific PROM [11]. The clinician discusses the KLIK ePROfile during the outpatient consultation with patients and/or parents in order to monitor well-being over time, detect problems at an early stage and provide tailored advice and interventions. Currently, more than 17,000 patients from 70 different patient groups (e.g., rheumatology, diabetes, oncology) have registered themselves on the KLIK website and around 1,000 clinicians (e.g., physicians, nurses, psychologists, social workers, physiotherapists, dieticians, and speech therapists) have been trained (around 800 active users) in the use of KLIK in daily clinical practice in > 20 different hospitals in the Netherlands [12] and 3 hospitals in the United Kingdom (www.klik-uk.org).

Nevertheless, implementing a PROM portal in clinical practice is a challenging process in which the interests of different stakeholders are involved [12, 13]. For a successful implementation, different determinants can be distinguished on the level of intervention characteristics, the clinician, the patient (and parent), and the socio-political context. In the past years, the intervention characteristics of the KLIK PROM portal have been evaluated repeatedly and adapted so that identified barriers for implementation for this determinant have been addressed [12, 13]. For example, PROMs are now available in multiple languages and KLIK has become an adaptable system to meet many individual wishes of the multidisciplinary teams [11]. However, little is known about barriers at the level of both clinicians and patients/parents. More insight is needed to fully understand the experienced barriers and to be able to optimize the KLIK PROM portal with regard to the wishes and needs of the user. Therefore, the aim of this study is to get more systematic insight into the experiences with KLIK from a clinician’s perspective.

## Methods

### KLIK implementation process

**Fig. 2:**
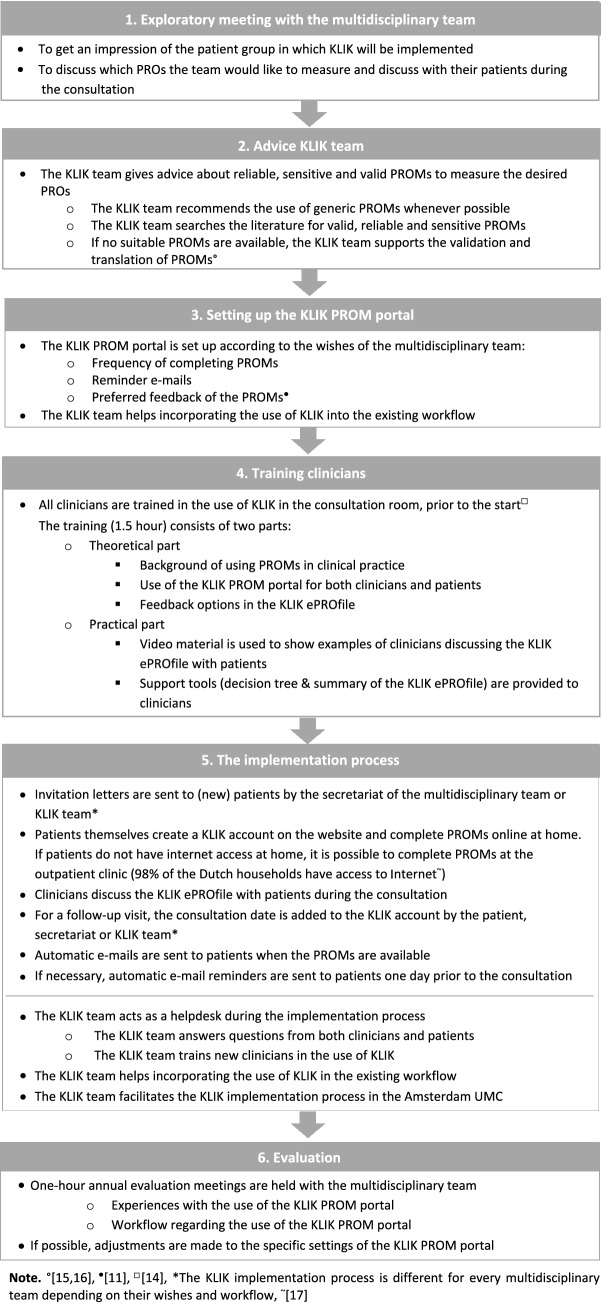
Overview of the KLIK implementation process for one multidisciplinary team. *Note* ^○^[15, 16], ^•^[11], ^□^[14], *The KLIK implementation process is different for every multidisciplinary team depending on their wishes and workflow, ~[17]

KLIK can be implemented for every multidisciplinary team (e.g., diabetes, dermatology) in health care [12]. The implementation process starts at request of a multidisciplinary team and is guided by the KLIK expert team (consisting of researchers with expertise in the field of PROMs and HRQOL research) of the Emma Children’s Hospital Amsterdam UMC through the following phases (Fig. 2):

1. The KLIK expert team has an exploratory meeting with the clinicians of the multidisciplinary team to get an impression of the patient group and the Patient Reported Outcomes (PROs) they would like to discuss in the consultation room.
2. The KLIK expert team gives advice about reliable, sensitive, and valid PROMs to measure the desired PROs. Whenever possible, PROMs with high reliability for specific populations and settings are selected to be able to use them on an individual level. However, sometimes the psychometric properties are not sufficient or unknown for the specific population, but no alternatives are available (e.g., in pediatrics, or in rare diseases).
3. The KLIK website is set up according to the wishes and workflow of the multidisciplinary team (e.g., frequency of completing PROMs, which reminder e-mails should be sent etc.). At this moment, over 300 PROMs have been built into the KLIK PROM portal. PROMs are offered to patients depending on age and patient group. Each member of the multidisciplinary team sees feedback of their preferred outcome measure set in a personal KLIK ePROfile.
4. Prior to the start of the implementation, all clinicians are trained in the use of KLIK in the consultation room. The 1.5 h training consists of a theoretical and a practical part. In the theoretical part, attention is paid to the definition of PROs and PROMs, the importance of discussing PROMs in the consultation room, and the use of the KLIK PROM portal including the different feedback options. In the practical part clinicians are trained in discussing the KLIK ePROfile with patients [14].
5. Throughout the implementation process, the KLIK expert team acts as a helpdesk for both clinicians and patients. For example, the KLIK expert team supports the integration of KLIK into the existing workflow of a multidisciplinary team and helps patients and/or parents to log into the KLIK website and complete PROMs.
6. As standard part of the KLIK implementation process, the KLIK expert team offers annual one-hour evaluation meetings to multidisciplinary teams to evaluate the use of KLIK in daily clinical practice and to identify and overcome barriers in the implementation process.

### Design and procedure

From February 2018 until August 2019, online evaluation questionnaires were sent out one week prior to each evaluation meeting. Reminder e-mails were sent to clinicians who had not completed the questionnaire one day before the meeting. The answers of the clinicians on the questionnaire on a team level provided a starting point for the evaluation meeting. Clinicians who had not completed the questionnaire prior to this meeting were asked to do so afterwards. This study has been approved by the Medical Ethics Committee of the Amsterdam University Medical Centers (Amsterdam UMC).

### Participants

Two hundred and forty-three team members (independent of their presence during the evaluation meeting) of 36 multidisciplinary teams in 14 hospitals that use KLIK were approached to participate in this study prior to a KLIK evaluation meeting. Multidisciplinary teams who use the KLIK PROM portal only for scientific purposes (6 multidisciplinary teams), where the implementation process started less than a year ago (N = 14) or teams that did not respond (N = 14) were not eligible. Supplement 1 provides an overview of the inclusion process.

### Measure

An evaluation questionnaire (Supplement 2) was developed to obtain the opinion of clinicians about the use of KLIK in daily clinical practice. The evaluation questionnaire was composed by four researchers of the KLIK expert team and reviewed by three nurses and one pediatrician. The questionnaire consisted of 20 closed questions (response options: three- and five-point Likert Scales, Visual Analogue Scales (VAS) and check boxes) and four mandatory open questions (a) advantages and (b) disadvantages of KLIK, (c) incentives for patients and (d) frequently heard reactions of patients about KLIK) regarding (1) overall satisfaction, (2) feeling competent to discuss PROMs, (3) use of KLIK during the consultation, (4) influence of KLIK on the consultation, (5) usability of the KLIK PROM portal, (6) satisfaction with PROMs and feedback, and (7) support of the KLIK expert team. There was room to add a comment or explanation with each question. Since every multidisciplinary team uses a different subset of PROMs and feedback options, not all questions in the domain ‘satisfaction with PROMs and feedback’ could by answered be all clinicians.

### Analysis

The Statistical Package for Social Sciences (SPSS) version 25.0 was used for descriptive statistics (percentages) to provide insight into the opinion of clinicians regarding KLIK and to study barriers and facilitators for the implementation process. Open questions of the evaluation questionnaire were analyzed qualitatively, by clustering the answers of all clinicians into main themes. This was done by two researchers (LT & HAvO) following the method for thematic analysis in Psychology [18]. Themes are ranked based on the number of times they have been mentioned by the clinicians (most often to fewest times).

## Results

### Participants

The online evaluation questionnaire was completed by 148 clinicians (61%), who were part of 36 different multidisciplinary teams from the following 14 different hospitals (Supplement 1): Emma Children’s Hospital (N = 57 participating clinicians), Amsterdam UMC locations VU Medical Center (N = 24) and Academic Medical Center (N = 4), Kidz & Ko – diabetes collaboration centers (N = 18), Reade (N = 8), University Medical Center Groningen (N = 7), Spaarne Hospital (N = 6), VieCuri Medical Center (N = 6), Zuyderland Medical Center (N = 5), Maasstad Hospital (N = 5), Kempenhaeghe epilepsy center (N = 3), Sophia Children’s Hospital (N = 2), Radboud University Medical Center (N = 2), and Wilhelmina Children’s Hospital (N = 1). Discipline and disease group of participating clinicians are shown in Table 1. On average, participating clinicians used KLIK for 3.3 years (range 0.2–8.8 years). Most participating clinicians were employed as medical doctor (N = 57), psychologist (N = 39), or nurse (N = 36), and multidisciplinary teams were divided into pediatrics (32 teams) and adult health care (4 teams).

**Table 1:** Characteristics of participants

	Participants (N = 148)
	N (% response-rate within discipline or group)
*Discipline*	
Medical doctor	57 (63.3)
Psychologist	39 (52.0)
Nurse	36 (66.7)
Dietitian	5 (71.4)
Physiotherapist	4 (100.0)
Social worker	3 (50.0)
Occupational therapist	2 (66.7)
Speech therapist	2 (100.0)
*Disease group*	
Diabetes (6 hospitals)	42 (63.6)
Juvenile Idiopathic Arthritis (2 hospitals)	12 (80.0)
Medical psychology (2 hospitals)	10 (52.6)
Sickle cell disease	9 (100.0)
Gender dysphoria	8 (27.6)
Coagulation diseases (4 hospitals)	7 (77.8)
Diagnostic Center Nutritional problems	6 (100.0)
Gastrointestinal diseases	6 (75.0)
Marfan syndrome	5 (100.0)
Neonatology follow-up	5 (71.4)
Spina Bifida	5 (55.6)
Cystic Fibrosis	4 (100.0)
Nephrology (2 hospitals)	4 (50.0)
Epidermolysis Bullosa	4 (44.4)
Surgery follow-up	4 (36.4)
Epilepsy	3 (75.0)
Human Immunodeficiency Virus	3 (50.0)
Congenital hand and arm disorders	2 (100.0)
Home Parenteral Nutrition	2 (66.7)
Metabolic diseases (2 hospitals)	2 (66.7)
Dermatology	2 (40.0)
Neurofibromatosis type 1	1 (100.0)
Muscle diseases	1 (50.0)
Endocrinology	1 (33.3)

#### Overall satisfaction

Clinicians (N = 147) reported an overall satisfaction with the KLIK PROM portal of *median* = 69, range 13–100, on a VAS ranging from 0 (not satisfied) to 100 (very satisfied). One clinician could not fill in the VAS due to technical problems.

#### Feeling competent to discuss PROMs

Almost all clinicians (89.9%) indicated that the KLIK training had prepared them sufficiently to use KLIK in daily clinical practice (8.1% neutral, 2% disagree). In addition, 85.8% of the clinicians felt competent to discuss the KLIK ePROfile with patients and/or parents in the consultation room (7.4% neutral, 6.8% disagree).

#### Use of KLIK during the consultation

Table 2 gives an overview of the use of KLIK reported by the clinicians. Most clinicians (70.3%) indicated they discuss the KLIK ePROfile (almost) always with patients and/or parents, 18.2% reported to discuss the KLIK ePROfile sometimes and 11.5% indicated to (almost) never discuss the KLIK ePROfile. Reasons for not discussing the KLIK ePROfile with patients and/or parents, as indicated by clinicians in the comments section, were lack of time, PROMs not completed, forgot to discuss, technical problems, no priority, no problems reported in the KLIK ePROfile, the KLIK ePROfile was discussed by another team member or KLIK was no longer part of standard care. Clinicians indicated they discuss the KLIK ePROfile at the start (42.6%), middle (37.8%), or end (19.6%) of the consultation. Clinicians estimated that they spend on average 15% of the consultation (broad range of consultation time; 10–50 min) on discussing the KLIK ePROfile and 85.8% of the clinicians were satisfied with this percentage.

**Table 2:** Scores on the domain ‘use of KLIK during the consultation’ (N = 148)

			(Almost) always (%)	Sometimes (%)	(Almost) never (%)
*Clinician*					
I discuss the KLIK ePROfile with patients/parents			104 (70.3)	27 (18.2)	17 (11.5)

			Start (%)	Middle (%)	End (%)
I discuss the KLIK ePROfile at the … of the consultation			63 (42.6)	56 (37.8)	29 (19.6)

			Median (range)		
On average, I spend % of the consultation on discussion of the KLIK ePROfile (N = 147)			15 (0–100)		

			Yes (%)	No, I need more time (%)	No, I need less time (%)
I am satisfied with the time I spent discussing the KLIK ePROfile			127 (85.8)	20 (13.5)	1 (0.7)

			Agree (%)	Neutral (%)	Disagree (%)
*About patients*					
All patients are invited to participate in the KLIK PROM portal			104 (70.3)	13 (8.8)	31 (20.9)

	100 (%)	75 (%)	50 (%)	25 (%)	0 (%)
I estimate that …% of patients/parents complete the PROMs	2 (1.4)	62 (41.8)	50 (33.8)	33 (22.3)	1 (0.7)

The majority of the clinicians (70.3%) invited all patients to participate in the KLIK PROM portal. Patients were not invited for the following reasons: absence of a chronic health condition, presence of a language barrier, a mental disability, illiteracy or not falling into a specific age range. In addition, clinicians mentioned they sometimes forgot to invite patients or they did not see it as their responsibility. 43.2% of the clinicians estimated that 75–100% of their patients and/or parents completed the PROMs. According to clinicians, reasons for not completing PROMs by patients were no Internet access, language barrier, forgetting, and loss of motivation.

#### Influence of KLIK on the consultation

According to 70.3% of the clinicians, their consultation improved by the use of the KLIK PROM portal (24.3% neutral, 5.4% disagree) and 60.1% of the clinicians detected problems in functioning of patients and/or parents sooner (33.8% neutral, 6.1% disagree). Reasons for not detecting problems sooner with the use of KLIK were that another team member discussed the KLIK ePROfile with patients and/or parents or that the clinician was already aware of the functioning of the patients. Half of the clinicians (48.6%) indicated that they thought patients and/or parents were satisfied with the use of KLIK, 45.3% of the clinicians indicated that they did not know and 6.1% of the clinicians indicated that they thought patients and/or parents were not satisfied. Reasons why patients were not satisfied according to clinicians were: *many questions* (time intensive, having to complete PROMs too often, repetition in questions), *practical problems* (no Internet, login problems) and/or *no motivation* (annoying, no added value).

Regarding the open questions (Table 3), main advantages of KLIK for clinicians were: *insight in patient’s functioning*, *improved communication*, *detecting problems*, *insightful feedback, patients being better prepared, easy to use*, *time saving,* and *clinician was better prepared.* Main disadvantages of KLIK for clinicians were: *low response-rate*, *takes time for clinician*, *irrelevant content of PROMs*, *complex procedure*, *technical aspects*, *no integration with Electronic Health Record (EHR)*, and *takes time for patients*. Table 3 shows the most important advantages and disadvantages of KLIK, expressed by clinicians.

**Table 3:** Advantages and disadvantages of KLIK and the use of PROMs, according to clinicians (N = 148)

	Examples
*Advantages of KLIK/PROM use*	
1. Insight in patient’s functioning	‘You quickly can get an impression of the things that are (not) going well’ ‘Monitoring the patient over time’ ‘Quick overview of how the patient is doing’
2. Improved communication	‘The KLIK ePROfile structures the consultation’ ‘It provides a starting point for the conversation on difficult topics’ ‘Makes it possible to go in depth more quickly’
3. Detecting problems	‘Problems are recognized earlier’ ‘It provides information about the disease/person that I would not have discovered otherwise’ ‘Standardized screening’
4. Insightful feedback	‘Graphs provide insight’ ‘Convenient that scores are calculated directly and automatically’ ‘Better overview of the results through traffic light colors and graphs’
5. Patients being better prepared	‘Provides patients the opportunity to think in advance about questions and concerns. They are not confronted with these during the consultation’ ‘Patients and parents talk to each other about items that matter’ ‘Patients think in advance about their own functioning and request for help’
6. Easy to use	‘User-friendly’ ‘Accessible’ ‘Completing PROMs at home is easier for patients/parents’
7. Time saving	‘The consultation is quicker’ ‘Saves time’ ‘As a clinician, it takes me less time than PROMs on paper’
8. Clinician was better prepared	‘Better and more targeted preparation of the consultation’ ‘Prior to the consultation, I have important information from patient and parents’ ‘Before the consultation, I already have an impression of the complaints’
*Disadvantages of KLIK/PROM use*	
1. Low response-rate	‘Patients often do not complete PROMs’ ‘Patients with problems, for whom KLIK adds value, rarely complete the questionnaires’ ‘Reminders are necessary for patients to complete PROMs’
2. Takes time for clinician	‘Extra time is needed to prepare the consultation’ ‘It takes time to discuss, since KLIK is not integrated into the EHR’ ‘Motivating patients to complete PROMs takes time’
3. Irrelevant content of PROMs	‘Not all questions are relevant for every patient’ ‘Patients misunderstand questions’ ‘Many questions’
4. Complex procedure	‘Patients lose username and password’ ‘PROMs are not easy to complete for parents with a cognitive disability or foreigners’ ‘Not all patients have access to Internet’
5. Technical aspects	‘It takes effort to log in’ ‘I do not receive an automatic message when patients have completed PROMs’ ‘I have to print the KLIK ePROfile, because we do not have computers in the consultation room’
6. No integration with EHR	‘The data from KLIK does not end up directly in the EHR’ ‘No integration with Epic©’ ‘Need to open a separate window, besides EHR’
7. Takes time for patients	‘Requires time investment of patients’ ‘Patients indicate that they sometimes spend a long time completing PROMs’ ‘Extra burden for busy parents’

According to clinicians, incentives for patients to use the KLIK PROM portal were: *insight in functioning* (reflection, awareness), *preparation for consultation* (time to think, conversation topics), *improved communication* (starting point for conversation, structure, comprehensive), *feeling heard* (being taken seriously, acknowledgement), *to be offered interventions in time* (signaling, intervene), and *empowerment* (involvement, request for help). Ten clinicians (6.8%) indicated that they do not know what the benefits for patients are.

#### Usability of the KLIK PROM portal

According to 71.6% of the clinicians, the KLIK portal is easy to use (19.6% neutral, 8.8% disagree) and 83.8% of the clinicians indicated that the KLIK portal has an attractive layout (15.5% neutral, 0.7% disagree).

#### Satisfaction with PROMs and feedback

In general, 64.9% of the clinicians were satisfied with the selected PROMs (Table 4). Reasons why clinicians were not satisfied with the PROMs were too many PROMs, PROMs are not suitable for every patient and not all PROMs are available in multiple languages. Regarding the feedback of answers of the PROMs, 80.4% of the clinicians were satisfied with the feedback in the overall KLIK ePROfile. In the KLIK ePROfile the individual items in traffic light colors (Fig. 1a) were viewed most frequently by the clinicians (84.7%). Of these traffic light colors, clinicians discussed the red answers most often with patients/parents (84.7%), followed by orange (58.4%) and green answers (34.3%). The graphs (scores over time resp. comparison with peers) are discussed by 47.4% resp. 33.6% of the clinicians. Clinicians thought that the traffic light colors of the KLIK ePROfile are most important (*median* = 72), followed by literal answers (*median* = 71) and graphs (*median* = 70) (Fig. 1b), reported on a VAS, ranging from 0 (not important) to 100 (very important).

**Table 4:** Scores on the domain ‘satisfaction with PROMs and feedback’

	N	Agree (%)	Neutral (%)	Disagree (%)
I am satisfied with the PROMs offered	134	87 (64.9)	36 (26.9)	11 (8.2)
I am satisfied with the feedback of:				
Overall KLIK ePROfile	148	119 (80.4)	26 (17.6)	3 (2.0)
Literal answers	148	112 (75.7)	33 (22.3)	3 (2.0)
Traffic light colors	137	115 (83.9)	19 (13.9)	3 (2.2)
Graphs (scores over time and comparison with peers)	137	105 (76.6)	25 (18.3)	7 (5.1)

		Literal answers (%) (N = 148)	Traffic light colors (%) (N = 137)	Graphs (%) (N = 137)
I look at the following parts of the feedback in the KLIK ePROfile (multiple answers possible)		124 (83.8)	116 (84.7)	97 (65.5)

		Literal answers			Graphs		
		Green answers (%)	Orange answers (%)	Red answers (%)	Comparison with peers (graph) (%)	Scores over time (graph) (%)	Other (%)
I discuss the following parts of the KLIK ePROfile (multiple answers possible)	137	47 (34.3)	80 (58.4)	116 (84.7)	46 (33.6)	65 (47.4)	24 (16.2)*

		Median (range)
I think the following parts of the feedback of the KLIK ePROfile are important:		
Literal answers	147	71 (35–100)
Traffic light colors	136	72 (11–100)
Graphs	136	70 (12–100)

^*^Other parts of the KLIK ePROfile that clinicians discuss with patients: open questions and changes in literal answers over time. A part of the clinicians does not discuss the KLIK ePROfile with patients

#### Support KLIK expert team

82.5% of the clinicians indicated to know where to ask their questions regarding the use of the KLIK PROM portal (10.1% neutral, 7.4% disagree) and 71.6% indicated that there is enough support from the KLIK expert team (25.7% neutral, 2.7% disagree).

## Discussion

This study provided insight into the experiences of clinicians with the use of the KLIK PROM portal in daily clinical care, at a group level. Overall, clinicians were satisfied with discussing PROMs in the consultation room via the KLK PROM portal. Clinicians indicated that discussing PROMs helps them to gain more insight into patient functioning, to improve the communication with patients, to detect psychosocial or physical problems, and to empower patients. These benefits are in line with previous effectiveness studies [3, 4, 6]. In addition, clinicians valued specific characteristics of the KLIK ePROfile, such as ease of use and the well-developed and insightful feedback. Regarding this feedback, clinicians mentioned they appreciated and looked at the individual item feedback in traffic light colors most often. This preference was also found in previous research on the feedback of the QLIC-ON Profile [19].

Although clinicians indicated that the KLIK training sufficiently prepared them to use KLIK in clinical practice, they also indicated that the training did not fully meet their needs. More explanation about the interpretation of PROM results and the use of cutoff scores would increase their sense of competence. In addition, a refresher course every few years would be desirable. For this reason, the KLIK expert team is now revising the KLIK training. More information and tips and tricks about the interpretation and communication of PROM results will be included.

Clinicians indicated that they do not always discuss the PROMs with patients and/or parents due to lack of time, technical problems or lack of clarity regarding the workflow. For some clinicians it is unclear which team member of the multidisciplinary team discusses the PROMs with patients and/or parents or who sends invitations. This indicates that continuous support with the implementation process and annual evaluation meetings with all team members of a multidisciplinary team remains necessary. Also, patients do not always complete PROMs prior to the outpatient consultation. Forgetting, loss of motivation or no Internet access were reasons from the clinicians’ perspective. In supporting the implementation process, a commonly heard argument from patients for not completing the PROMs is that the clinician does not discuss the PROMs during the consultation. This indicates how important it is for clinicians to discuss the PROMs with patients and/or parents. In addition, it was mentioned that for patients (or parents) with low health literacy skills and for non-native Dutch speakers it is sometimes difficult to complete the PROMs. Although the most frequently used generic PROMs in KLIK are available in multiple languages, this is not the case for all PROMs. When compiling the PROMs outcome sets with the multidisciplinary teams, more attention should also be paid to the needs of non-native Dutch speakers and patients with low health literacy skills.

Clinicians reported several main barriers for using PROMs via the KLIK portal. The first one is a lack of integration between KLIK and the EHRs. Opening a separate website to view the KLIK ePROfile is an added operation for clinicians, with the consequence that the KLIK ePROfile is sometimes not discussed with patients and/or parents. Therefore, in September 2019 a front-end integration with the two most often used EHRs in the Netherlands, Epic© and HiX© was realized in four hospitals. Clinicians can now view the KLIK ePROfile via the EHR, which increases the user-friendliness and makes it a better fit into the clinical workflow.

Second, clinicians indicated that they are not always satisfied with the content of PROMs. Reasons were mostly focused on the burden of completing PROMs for patients, such as a long completion time, many repetitions in questions and irrelevant questions. These challenges with PROMs correspond with previous research [20]. To address these problems, the National Institute of Health (NIH) developed the Patient-Reported Outcome Measurement Information System (PROMIS) [21, 22]. PROMIS consists of various dynamic item banks (each measuring a separate construct) that can be administered through computerized adaptive testing (CAT) [20, 23]. By using a CAT, questions are offered based on the person’s previous answer. In this way, patients and/or parents only have to answer a few questions per PROMIS construct to get a reliable score. As a result, the burden for patients and/or parents can be reduced [24]. Since November 2019, it is possible to administer the PROMIS item banks via KLIK, by linking KLIK with the Dutch Assessment Center. To realize this, the PROMIS item banks were translated and validated in the Netherlands [11, 16].

Third, clinicians mentioned that the use of PROMs is time intensive. Clinicians indicated that it takes more time to prepare themselves for the consultation and to discuss the PROMs in the consultation room. This is a remarkable finding, since previous research has shown that the use of the QLIC-ON Profile did not lengthen the consultation [9]. In addition, clinicians who are responsible for inviting patients for the KLIK PROM portal indicated that it takes a lot of effort to motivate patients to complete PROMs. A case manager that supports the KLIK implementation would be helpful.

There were a few limitations to this study. First, not all clinicians that use KLIK in the consultation room have been included in this study, because not all multidisciplinary teams were open to an evaluation meeting despite the importance for the implementation process. However, the experiences of clinicians from different disciplines, working with various disease groups in multiple hospitals and different outcome measure sets were included. Second, completing the VAS of the domains ‘overall satisfaction’ and ‘satisfaction with PROMs and feedback’ was not always possible when using a tablet. For these clinicians, it was not possible to move the bar to the desired position, causing a score around 50. Unfortunately, it could not be traced who had had this problem and therefore the results of these questions should be interpreted carefully. Third, the question ‘I am satisfied with the PROMs offered’ was not always understood by the clinicians. Prior to this question, there was a question about specific PROMs. The explanations showed that some clinicians referred to the specific PROMs when answering this question. That is why the answers to this question of 14 clinicians were not included. Fourth, due to the used method, this study provides no insight into the actions clinicians take with regard to the completed PROMs. In addition, no questions were asked about how clinicians use the information from the completed PROMs in daily clinical care. Therefore, recommendations for future PROM implementation research are to gain more insight into the actions of clinicians with regard to the discussed PROMs and how this can lead to more patient-centered care. The use of video observations in the consultation room may provide this information.

To conclude, the KLIK PROM portal is a valuable tool for clinicians to systematically monitor the functioning of their patients in clinical practice, so that extra support can be offered when needed. Overall, clinicians were enthusiastic about the feedback and user-friendliness of the KLIK PROM portal and the added value of using PROMs in clinical practice. However, some challenges and barriers were also identified. Therefore, a next step is to address the mentioned feedback points in the KLIK portal to improve the user-friendliness. Also, the perspective of the other user group, the patients and parents, is needed to further adapt the KLIK PROM portal to their wishes. Therefore, a similar study will be performed in the near future evaluating the KLIK PROM portal from the patients’ perspective, with the ultimate goal to further optimize the KLIK PROM portal and to improve the quality of health care.

## Electronic supplementary material

Below is the link to the electronic supplementary material.

Supplementary file1 (DOCX 79 kb)

Supplementary file2 (DOCX 19 kb)
