# Supplementary material for: Clinicians’ perspective on the implemented KLIK PROM portal in clinical practice
Source: Qual Life Res. 2020 May 28;30(11):3267–77. doi: 10.1007/s11136-020-02522-5 (PMC8528749; doi:10.1007/s11136-020-02522-5)
Supplement: Supplementary file 1 — Supplementary file1 (DOCX 79 kb) [file 11136_2020_2522_MOESM1_ESM.docx]

Supplement 1 – Flow diagram of participating hospitals, multidisciplinary teams and clinicians
