# Supplementary material for: Clinicians’ perspective on the implemented KLIK PROM portal in clinical practice
Source: Qual Life Res. 2020 May 28;30(11):3267–77. doi: 10.1007/s11136-020-02522-5 (PMC8528749; doi:10.1007/s11136-020-02522-5)
Supplement: Supplementary file 2 — Supplementary file2 (DOCX 19 kb) [file 11136_2020_2522_MOESM2_ESM.docx]

**Evaluation questionnaire KLIK**

**Overall satisfaction**

1. In general, how satisfied are you with the use of KLIK?

Not satisfied at all .................................... very satisfied *(VAS 0-100)*

**Feeling competent to discuss PROMs**
 Strongly disagree Disagree Neither disagree Agree Strongly agree
 nor agree

1. The KLIK training has prepared me sufficiently to start using KLIK ❑ ❑ ❑ ❑ ❑
    Explanation:
2. I feel competent to discuss the KLIK ePROfile with patients/parents ❑ ❑ ❑ ❑ ❑
    Explanation:

**Use of KLIK during the consultation**

1. I discuss the KLIK ePROfile with patients/parents ❑ Always ❑ Usually ❑ Sometimes ❑ Almost never ❑ Never
    Explanation:

*When: usually, sometimes, almost never and never, then:*

4a. The reason why I do not always discuss the KLIK ePROfile is:
*Open answer*

5. I discuss the KLIK ePROfile at the … of the consultation ❑ Start ❑ Middle ❑ End
 Explanation:

6. On average, I spend …. % of the consultation on discussing the KLIK PROfile
❑ I am satisfied with this
❑ I would like to spend more time on discussing the KLIK PROfile, because …
❑ I would like to spend less time on discussing the KLIK PROfile, because …

*Patient* Strongly disagree Disagree Neither disagree Agree Strongly agree nor agree

1. All patients are invited to participate in the KLIK PROM portal ❑ ❑ ❑ ❑ ❑
    Explanation:
   *When: strongly disagree, disagree, neither disagree, nor agree:*

7a.That not everyone is asked to participate is based on:
*Open answer*

1. I estimate that … % of the patients/parents complete the PROMs ❑ 100% ❑ 75% ❑ 50% ❑ 25% ❑ 0%
    Explanation:

**Influence of KLIK on the consultation**
 Strongly disagree Disagree Neither disagree Agree Strongly agree
 nor agree

1. KLIK improves my consultation ❑ ❑ ❑ ❑ ❑
    Explanation:
2. With the use of KLIK, I detect problems sooner ❑ ❑ ❑ ❑ ❑
    Explanation:
3. Advantages of KLIK are:
    *Open answer*
4. Disadvantages of KLIK are:
    *Open answer*

*Patient* Strongly disagree Disagree Neither disagree Agree Strongly agree nor agree

1. I think that patients/parents are satisfied with the use of KLIK ❑ ❑ ❑ ❑ ❑
    Explanation:
2. I think that incentives for patients/parents to use KLIK are:
   *Open answer*
3. The most frequently heard reactions of patients/parents about KLIK are:
    *Open answer*

**Usability of the KLIK PROM portal** Strongly disagree Disagree Neither disagree Agree Strongly agree
 nor agree

1. The KLIK PROM portal is easy to use ❑ ❑ ❑ ❑ ❑
    Explanation:
2. The KLIK PROM portal has an attractive lay-out ❑ ❑ ❑ ❑ ❑
    Explanation:

**Satisfaction with PROMs and feedback** Strongly disagree Disagree Neither disagree Agree Strongly agree
 nor agree

1. I am satisfied with the PROMs offered ❑ ❑ ❑ ❑ ❑
   Explanation:

Strongly disagree Disagree Neither disagree Agree Strongly agree
 nor agree

1. I am satisfied with the feedback of:
   1. Overall KLIK ePROfile ❑ ❑ ❑ ❑ ❑
   2. Literal answers ❑ ❑ ❑ ❑ ❑
   3. Traffic light colors ❑ ❑ ❑ ❑ ❑
   4. Graphs (scores over time and comparison with peers) ❑ ❑ ❑ ❑ ❑
      Explanation:
2. I look at the following parts of the feedback in the KLIK ❑ Literal answers ❑ Traffic light colors ❑ Graph ❑ Otherwise
   ePROfile (multiple answers possible)
    Explanation:
3. I discuss the following parts of the KLIK ePROfile (multiple answers possible)
   ❑ Green answers
   ❑ Orange answers
   ❑ Red answers
   ❑ Comparison with peers (graph)
   ❑ Scores over time (graph)
   ❑ Other…..
4. I think the following parts of the feedback of the KLIK ePROfile are important:
   1. Literal answers Not important at all ………………………………Very important *(VAS 0-100)*
   2. Traffic light colors Not important at all ………………………………Very important *(VAS 0-100)*
   3. Graphs Not important at all ………………………………Very important *(VAS 0-100)*

**Support KLIK expert team**
 Strongly disagree Disagree Neither disagree Agree Strongly agree
 nor agree

1. I know where I can ask my questions regarding the KLIK PROM portal ❑ ❑ ❑ ❑ ❑
    Explanation:
2. There is sufficient support from the KLIK team when I need it ❑ ❑ ❑ ❑ ❑
    Explanation:
